# Pineal germinoma in a young adult: A case report

**DOI:** 10.1002/cnr2.1611

**Published:** 2022-03-28

**Authors:** Lissett Jeanette Fernández‐Rodríguez, Xavier Maldonado‐Pijoan

**Affiliations:** ^1^ School of Medicine Universidad Privada Antenor Orrego Trujillo La Libertad Peru; ^2^ Department of Medicine Hospital de Alta Complejidad Virgen de la Puerta La Esperanza La Libertad Peru; ^3^ Oncology and Radiotherapy Service Hospital Universitario Vall d'Hebron Barcelona Spain

**Keywords:** case report, germinoma, intensity‐modulated radiotherapy, neurosurgery, pineal gland

## Abstract

**Background:**

Intracranial germinomas (GN) are rare cancers that primarily affect children, making them rarer still in adults. Standard treatment for this neoplasm includes neoadjuvant chemotherapy (NC) followed by radiotherapy (RT) or RT at a higher dose and larger field. These recommendations are based on studies focused mostly on children; it is currently unclear whether this treatment is applicable to adults.

**Case:**

We present a case of a 23‐year‐old adult male with no underlying pathologies, drug allergies, or family history of cancer, who presented for medical evaluation with blurred vision, diplopia, forgetfulness, and weight loss starting 3–4 months before the evaluation. Clinical examination indicated Parinaud's Syndrome. Magnetic resonance imaging (MRI) and computed tomography (CT) revealed a pineal tumor with ependymal dissemination in both lateral ventricles, which was causing obstructive hydrocephalus. The patient had surgery consisting of ventriculostomy, Holter shunt insertion, cisternal ventricular intubation, and cisterna magna anastomosis to improve ventricular drainage. Pathology confirmed pineal germinoma. Cerebrospinal fluid cytology and MRI of the axis were negative. Four cycles of NC were given to the patient (carboplatin, etoposide, and ifosfamide), with reduced dosage. Once a partial volumetric response was confirmed, whole‐ventricular radiotherapy (WVR) was initiated with a total tumor bed dose of 45 Gy over 25 sessions in 5 weeks. Optimum clinical results were observed, and no short‐term (<90 day) radiation toxicity was observed. The patient was able to resume his normal activities soon after treatment. Follow‐ups over 2 years post‐surgery indicated continued control of the lesion and absence of symptoms except for mild diplopia.

**Conclusion:**

Although this is a case report, these data suggest that a reduced NC course and WVR may effectively treat adult GN. This protocol likely decreases the risk of undesirable NC and RT secondary effects, while providing excellent local control; however, using a narrower RT field is not recommended.

## 1 INTRODUCTION

Primary central nervous system germ cell tumors (CNS‐GCT) are a rare form of neoplasm that are likely caused by germ cells trapped in midline locations during fetal development.^1^ They are most found in children, with approximately 90% of cases diagnosed in patients less than 20 years old. CNS‐GCT comprises about 0.5%–1% of all primary brain tumors diagnosed in children and young adults, giving an incidence of about 0.1 per 100 000 person‐years.^2^ Some studies show that the prevalence of CNS‐GCT is higher in Asia and Europe than in the USA,^1^ while others have not found this regional variation.^1^, ^2^

The most common type of CNS‐GCT is the germinoma (GN), which comprises about 75% of all CNS‐GCT. As with other CNS‐GCT, most patients are diagnosed before 20 years of age and are predominately male. GN is most frequently found in the brain midline, the pineal gland, and/or suprasellar regions,^3^ where it slowly spreads into adjacent tissues and the subarachnoid space, following cerebrospinal ducts.^4^ The symptoms of GN are related to the size and location the tumor, which frequently causes endocrine disfunction, intracranial hypertension, and visual alterations, such as diplopia and Parinaud's syndrome in 75% of cases.^5^, ^6^, ^7^

The diagnosis of GN includes evaluation of clinical symptoms, analysis of oncoprotein in blood and/or cerebrospinal fluid (CSF), medical imaging, and histopathology.^1^ In pure GN, alpha‐fetoprotein (AFP), and beta‐human chorionic gonadotropin (BHCG) levels in serum and CSF are usually lower than what is found in patients with other intracranial GCTs. GN cells strongly and diffusely express tyrosine‐protein kinase KIT, CD117 (c‐KIT), octamer‐binding transcription factor 4 (OCT4), and placental alkaline phosphatase (PLAP).^1^, ^3^, ^8^, ^9^ GNs appear as solid masses that may include cysts on magnetic resonance images. They are often observed as isointense or high‐intensity gray matter in T1 and T2‐weighted images with intense and homogenous enhancement in post‐contrast, where a typical butterfly shape can be observed. Reduced diffusion can be observed due to its highly cellular nature. Computerized axial tomography (CT) imaging is also helpful in diagnosis, as it can detect the presence of pineal calcifications that are frequently present in GN.^10^

When treated, GN has an excellent prognosis, with 5‐year progression‐free survival rates of more than 90%; some studies in children have seen survival at 100%.^1^ The current standard treatment protocol is a combination of neoadjuvant chemotherapy (NC), usually including etoposide, ifosfamide, and a platinum‐based agent coupled with whole‐ventricular radiotherapy (WVR; 20–24 Gy) followed by a 12–16 Gy boost to the tumor bed. Given the range of evaluated treatment options, a balance must be struck between the elimination of the tumor and damaging functional tissue.^1^

The overall rarity of GN and its predominance in children leave gaps in knowledge on how to treat these tumors in adults. Nearly all studies on GN treatment and outcome are based on pediatric cases or make no distinction between children and adults, which skews them toward children. Therefore, current standard treatment procedures have been developed with children in mind, but it has been questioned whether these standards are applicable to adults.^11^, ^12^

To address some of these gaps in knowledge, we present a case of a 23‐year‐old adult that was diagnosed with GN and treated according to the recommended protocol for children, but with a slightly higher dose of radiation and a reduced NC dosage.^1^

## 2 CASE

A 23‐year‐old male from Barcelona presented with symptoms of blurred vision, diplopia, and memory and weight loss for 3–4 months. The patient had no pathological antecedents of interest, family history of cancer, or known drug allergies.

**FIGURE 1:**
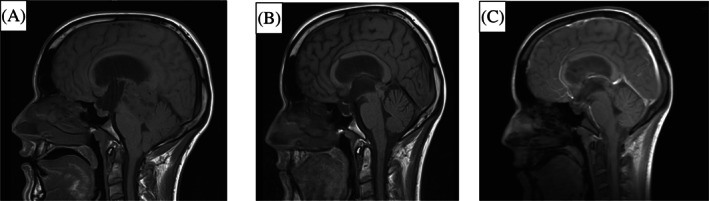
MRI T2 images of the case evolution. (A) Moment of diagnosis, (B) post NC, and (C) post WVR. NC, neoadjuvant chemotherapy; WVR, whole‐ventricular radiotherapy

Physical examination of the patient revealed Parinaud's syndrome. CT revealed the presence of a solid cystic lesion measuring 36 × 38 × 39 mm in the pineal region with hyperdense areas near the septum and lateral ventricles. Triventricular hydrocephalus was also observed. Cranial MRI indicated the presence of a 37 × 33 × 47 mm pineal tumor with ependymal spread in both lateral ventricles, causing obstructive hydrocephalus. Both tomograms were suggestive of GCT (Figure 1).

Two days after presenting at the hospital, the patient was admitted to surgery. The patient was placed under general anesthesia in a supine position with orotracheal intubation, with the head in a neutral position and immobilized with a Mayfield–Kees skull fixation apparatus. The initial incision was centered 12.5 cm from the nasion and 2.5 cm from the midline. Tissue was dissected by planes, and the skull was opened by a right front trephine hole. An external ventricular drain was inserted, and CSF was collected for analysis. An endoscope was inserted to observe the right lateral ventricle. The third ventricle was accessed through the right foramen of Monro. The foramen was widened by premamillary perforation and held open with a Fogarty balloon until the adequate connection between the two ventricles was achieved. A pink, cottony tumor lesion was observed in the anterior third of the third ventricle floor in front of the perforation. Two samples for histopathology were obtained. A tumor was also observed on the right lateral wall of the third ventricle, which had cystic characteristics and apparent ependymal preservation. Two samples were taken from this lesion. All samples were preserved in formalin for histopathological analysis. Finally, a septostomy was performed by removing the endoscope and inserting and fixing a styletless external ventricular drain. This procedure revealed an additional lesion in the anterior third of the septum. The surgery proceeded without incident with no hemorrhaging.

CSF analysis results were negative for malignant cells and contained the tumor markers BHCG at 10 IU/L and AFP < 1.3 ng/ml. Histopathological examination of the lesion biopsies indicated pineal GN, as the cells were round and resembled primitive germ cells with large vesicular nuclei, prominent nucleoli, prominent glycogen‐rich clear cytoplasm, lymphocyte infiltration, and infiltration cells along the fibrovascular septa. Cells of the lesions expressed c‐KIT, OCT4, and PLAP in an intense and diffuse manner. Given this information, the patient was diagnosed with pure pineal germinoma staged at M0.

It was recommended for the patient to undergo four cycles of NC followed by WVR, according to current treatment guidelines.^1^ NC (Table 1) was initiated 16 days post‐surgery. Twenty‐six days after the first cycle of NC, the patient evaluation revealed toxicity in the form of neutropenic fever, bleeding gums, weakness, thrombocytopenia, and Grade 4 neutropenia. To avoid these symptoms in subsequent rounds, the NC dose was reduced by 20%. The second round of NC began 47 days post‐surgery, but the patient later presented with Grade 1 thrombocytopenia, requiring a pause in the third planned round of chemotherapy. The planned final course of NC was initiated 92 days post‐surgery.

**TABLE 1:** Recommended NC treatment protocol

Day	Chemotherapy agent	Dose
1	Carboplatin	AUC: 5 (mg/ml) min
	Etoposide (VP‐16)	100 mg/m^2^
2	Ifosfamide	1800 mg/m^2^
	Etoposide (VP‐16)	100 mg/m^2^
3	Etoposide (VP‐16)	100 mg/m^2^
4	Ifosfamide	1800 mg/m^2^

Abbreviation: NC, neoadjuvant chemotherapy.

The external ventricular drain was removed and follow‐up cranioaxial MRI revealed a reduction in tumor size and a slight improvement in supratentorial ventriculomegaly without signs of ependymal activity or complications (Figure 1). There was no evidence of metastatic spread of the tumor to the spine.

**FIGURE 2:**
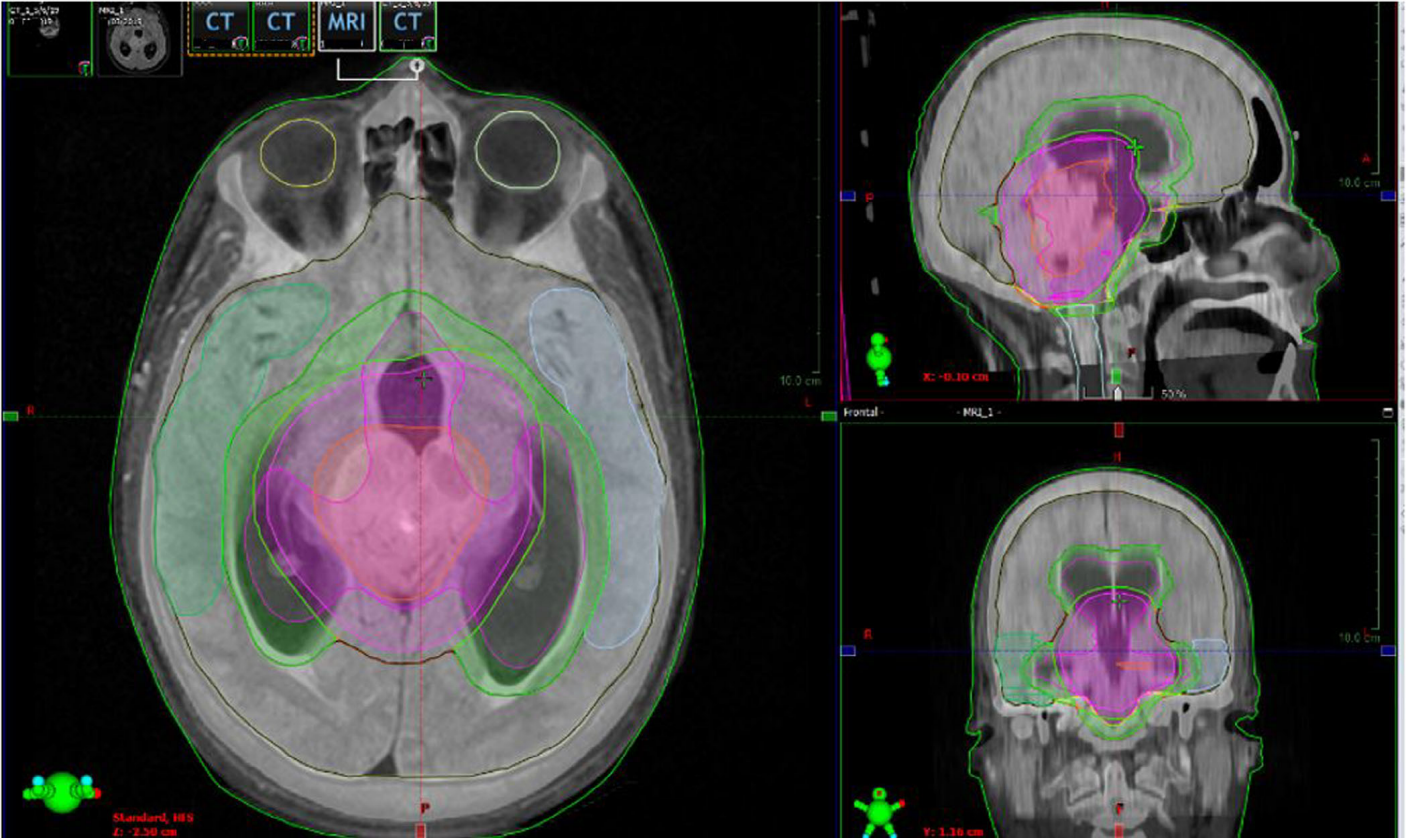
Radiotherapy planning process: computed tomography without contrast merged with the initial MRI

**FIGURE 3:**
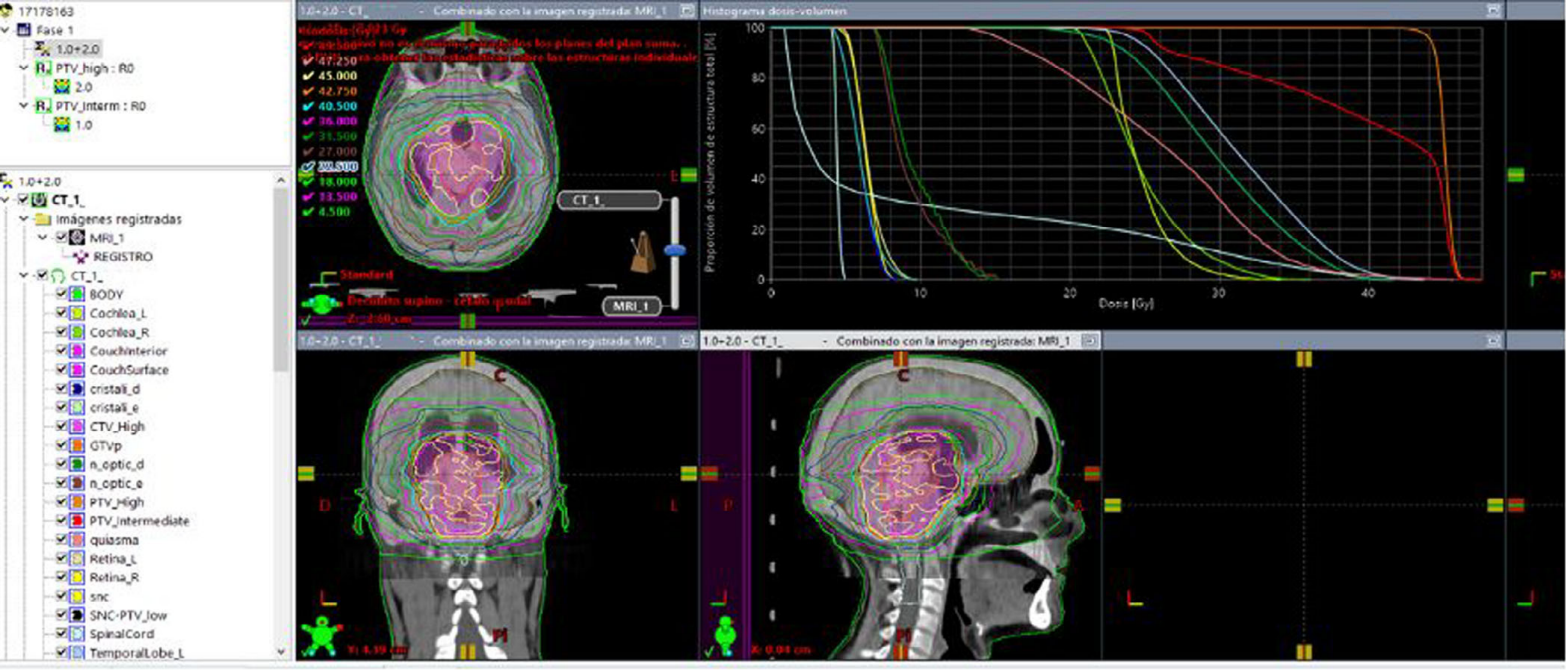
Radiotherapy dose estimation

Radiotherapy simulation was completed during NC treatment to develop a treatment plan (Figure 2). Planning included calculating radiation dose to different organs at risk (Table 2). Most organs at risk received less than half the dose constraint. As suggested by the simulation, RT using intensity‐modulated radiation therapy (IMRT) at 6MV was started 134 days post‐surgery with 13 WVR sessions at a dose of 1.8 Gy/session to the entire ventricle. A subsequent boost to the tumor bed consisted of 12 sessions at a dose of 1.8 Gy/session. The total dose was 45 Gy in 25 sessions over 5 weeks. WVR and radiation dose followed current treatment guidelines (Figure 3).^1^ During RT, the patient's vision improved clinically, and acute neurotoxicity was absent, but radiation induced G1 conjunctivitis was observed.

**TABLE 2:** Calculated dose to organs at risk with standard‐dose constraints

Organ at risk	Calculated average dose (Gy)	Dose constraints (Gy)^13^, ^14^, ^15^
Left cochlea	24.77	<45.00
Right cochlea	24.82	<45.00
Right temporal lobe	29.79	1 cm^3^ < 70.00
Left temporal lobe	31.44	1 cm^3^ < 70.00
Right eye	6.01	<20.00 in 100%
Left eye	5.92	<20.00 in 100%
Optic chiasma	26.37	<56.00
Right optic nerve	9.30	<56.00
Left optic nerve	9.65	<56.00
Left lens	4.41	<6.00
Right lens	4.46	<6.00

A post‐RT MRI (204 days post‐surgery) was compared to the post‐NC MRI, with the former showing a discrete decrease in the size of the tumor located in the pineal region. The tumor had a polylobulated morphology and had a heterogenous MRI signal with hypointense foci in the heme sequence in relation to calcifications and hemosiderin debris (Figure 1). A reduction in tumor size was observed between the post‐NC and post‐RT scans (from 19 × 15 × 18 mm, 2.5 ml to 18 × 12 × 17 mm, 1.8 ml, anterior/posterior × transverse × craniocaudal). At this point, it was recommended that the patient receive regular follow‐ups with MRI monitoring.

A T1 and T2 MRI follow‐up 659 days post‐surgery revealed encouraging results, when compared to previous brain MRIs. There was a stability in the size, signal characteristics, and MRI sequence behavior of the tumor remnants. The pineal tumor remnant had a polylobulated morphology and heterogenous resonance signal, which is associated with aqueductal stenosis and mild retraction of the posterior margin of the mesencephalic tegmentum. However, the permeability of the ventriculocystostomy and ventricular system did not change. Additionally, the arachnoid grooves were present in both cerebral convexities. There were no other alterations in the encephalic parenchyma, except a small path present on the right frontal lobe due to the ventricular drain. From these findings, the patient was shown to have a stable case. The patient stated that he was pleased with the treatment and was able to return to his normal activities.

An additional follow‐up 849 days after surgery revealed that the patient was asymptomatic except for diplopia. An MRI image revealed that the patient has stabilized and there were no new developments. Despite the diplopia, the patient was pleased with the treatment and was able to continue his normal activities.

## 3 DISCUSSION

We present a rare case of a 23‐year‐old male diagnosed with M0 primary pineal GN. The tumor was in the brain midline, where GN is usually found^1^, ^3^ and the patient suffered from common symptoms, such as weight loss, blurred vision, and memory problems. Parinaud's syndrome, caused by compression of the midbrain quadrigeminal lamina was also observed.^16^ Because of these neurological symptoms, the patient was admitted for emergency treatment. Computed tomography and MRI confirmed the presence of a mass that could explain the symptoms, which were partially relieved by laparoscopic ventricular drainage.

Analysis of CSF obtained from surgery revealed BHCG and AFP levels consistent with those of non‐secreting GCT, like germinoma, although germinoma cannot be definitively confirmed from these levels alone. Recommended thresholds for ruling out non‐secreting GCT in CSF are 50–100 IU/L for BHCG and 10–50 ng/ml for AFP. A more recent study based on pathology revised these levels to 8.2 IU/L for BHCG and 3.8 ng/ml for AFP to detect secreting GCT with high specificity, but lower sensitivity. The values determined here slightly exceed the recently recommended threshold for BHCG, but this threshold was designed to select for secreting tumors rather than eliminate non‐secreting tumors.^17^ Pathological anatomy was therefore needed to confirm germinoma; cells in the surgical biopsies strongly and diffusely expressed c‐KIT, OCT4, and PLAP.^1^, ^3^, ^8^, ^9^ In the tumor biopsies, we observed cells that resemble primitive germ cells as well as leukocyte infiltration. These observations are consistent with a GN diagnosis.^3^ Germinoma is more susceptible to radiation and chemotherapy treatment than other GCT, so a treatment plan that uses both was indicated.^1^

Several studies (Table 3) with differing treatment protocols have been conducted to propose or improve treatment recommendations for GN. Craniospinal RT has been shown to successfully treat GN. This mode is supported by several studies, including the MAKEI 83/86/89,^31^ SIOP CNS GCT 96,^25^ and others, which showed overall and event‐free survival more than 90%.^27^, ^30^ A small study on adults with germinoma had a similar outcome with an RT‐only approach.^28^ These studies also suggest that irradiation of wide regions of the brain with doses of 40–50 Gy has minimal long‐term toxicity and significantly decreases the risk of relapse.

**TABLE 3:** Comparison of different treatment protocols for intracranial germinoma and related cancers

Study	Methods	Results	Interpretation
This work	NC: 3 cycles of CB, ET, and/or IFO RT: 23.4 Gy whole ventricular + tumor bed boost to 45 Gy	Recovery of one patient	Largely untested but likely a good balance between elimination of the cancer and collateral damage
Bartels et al. (2020)^18^	NC: 4 cycles of CB and ET RT: WVR 18 Gy + 12 Gy tumor bed boost	Estimated 3‐year PFS was 94.4% (74 subjects)	Reduction in RT dose can be based on NC response
Li et al. (2020)^19^	NC: IFO, ET, cisplatin (2 cycles) RT: Focal radiotherapy, WVR, WBR + boost, or CSI + boost; most ≥40 Gy NC: 2 cycles as above	Estimated 5‐year disease‐free survival and OS were 96.7% and 97.3%	Focal radiotherapy has high risk for GN relapse, other RT types gave better results
Byun et al. (2020)^20^	Review of different treatments, including RT and NC + RT	NC alone has a high risk of relapse, as does narrow‐field RT. Adding NC to wide field RT seems to have minimal benefit	Wide field RT‐only therapy can cure GN at a high rate
Fetcko and Dey (2018)^1^	NC: 4 cycles of ET, IFO, CB/cisplatin RT: WVR 20–24 Gy + tumor bed boost of 12–15 Gy	Localized GN is highly treatable and has good prognosis	Recommended treatment based on multiple references
Fu et al. (2017)^21^	Comparison of RT only and NC + RT	Groups had different outcomes depending on follow‐up time	Both RT and NC + RT have good outcomes. NC + RT has better initial outcomes, but this is eliminated at 5 years
Leung et al. (2017)^22^	RT: primary tumor total dose of 54 Gy, 37 Gy to the whole ventricles (IMRT)	Patient free of disease with no adverse effects	IMRT can spare normal tissues and may reduce neurocognitive side‐effects
Krueger et al. (2016)^23^	RT only: cranial‐spinal axis 25.4 Gy, WBR 36 Gy, tumor location boost 50.4 Gy to the prominent midline and ventricular regions	Patient was asymptomatic 1 year after treatment	RT‐only protocol can have desirable results for GN with diffuse subependymal spread
Joo et al. (2014)^24^	NC: two cycles cisplatin and ET (other 2‐cycle regimens included) RT: CSI, WBR/WVR, Focal RT, 28–46 Gy to the tumor	Radiotherapy field was significantly associated with recurrence‐free survival, with CSI having the highest, at 95%	Patients showing complete response with NC are suggested to receive WBR/WVR
Calaminus et al. (2013)^25^	Craniospinal RT 24 Gy + boost 16 Gy versus induction CB/ET alt ET/ifos then 40 Gy IFRT	Improved PFS with CSI in localized germinoma 5‐year PFS 97% versus 88% (relapses in ventricles) 5‐year EFS ~ 90% and OS ~ 95%, not different	Localized germinoma can be treated with reduced dose CSI or induction chemo followed by focal RT, though PFS does favor CSI
Nitta et al. (2013)^26^	NC: cis‐diamminedichloroplatinum (ii) and ET RT: Focal RT, 24 Gy	Complete recovery of two brothers	Largely untested but promising
Alapetite et al. (2010)^27^	NC: CB, ET, IFO RT: Tumor bed 40 Gy	Excess of periventricular relapses	Suggests using ventricular field radiation to decrease relapse
Foote et al. (2010)^28^	RT: CSI of 25 Gy with local boost to 40 Gy	All patients alive after 10.9 years median follow‐up, no relapses	For adult intracranial GN, low‐dose CSI RT with local boost is highly effective with minimal morbidity
Jensen et al. (2010)^29^	Comparison of NC + RT or RT only: NC + RT: platinum‐based NC + 30.6 Gy to local fields, RT only: ~50 Gy to local fields	Less progression in the RT only group when larger fields were irradiated. Similar but not statistically significant results for NC + RT	Adding NC to RT for appropriate patients reduced GN relapse. Tumor control is improved when larger fields were irradiated
Ogawa et al. (2004)^30^	Various regimes	10‐year actuarial OS was 90%	No patients dosed with less than 55 Gy developed apparent neurocognitive disfunction. RT was a curative treatment for GN. Total dose of 40–50 Gy to appropriate treatment fields was effective in preventing intracranial relapse
Bamberg et al. (1999)^31^	RT: Craniospinal axis: 36 or 30 Gy followed by 15 or 15 Gy to the tumor region	5‐year relapse‐free survival 91.0%	Craniospinal RT at decreased dose levels is effective, further attempts to reduce dose are justified
Chao et al. (1993)^32^	RT: whole brain or tumor with margin (median dose 36 Gy), with or without the boost of 10–20 Gy	Relapse‐free survival was 100% at 2 years and 86% at 5 years	RT is effective in controlling germinoma

Abbreviations: CB, carboplatin; CSI, cerebrospinal radiation; EFS, event‐free survival; ET, etoposide; GN, germinomas; IFO, ifosfamide; IMRT, intensity‐modulated radiation therapy; NC, neoadjuvant chemotherapy; OS, overall survival; PFS, progression‐free survival; RT, radiotherapy; WBR, whole‐brain radiotherapy; WVR, whole‐ventricular radiotherapy.

Although effective, concerns over long‐term toxicity of craniospinal RT led to research on narrowing the irradiation field.^1^, ^3^, ^11^ This is especially important to consider as GN patients are generally young and follow‐up times of these studies are generally a small proportion of the expected life span of the patient. Reducing the field to whole‐brain radiotherapy (WBR) can be associated with a subacute and delayed decline in memory and other cognitive functions.^22^

A further reduction to WVR by IMRT and three‐dimensional conformal radiation therapy (3DCRT) was also evaluated.^32^ Although IMRT is associated with an increased dose of radiation to peripheral areas of the body, it better preserves the normal tissue of the CNS, when compared to either 3DCRT or WBR.^33^ In another study, IMRT was compared to proton beam therapy. This study showed that proton therapy provides a superior coverage of the target tissue and better preservation of normal tissue, such as the temporal lobes and hippocampus.^8^, ^34^ However, very little information is available regarding the long‐term toxicity of this technique.^35^

Restricting the field beyond whole ventricles significantly and consistently increases the risk of relapse.^19^, ^24^, ^25^, ^27^, ^30^, ^31^, ^36^ GN patients treated with highly localized doses in focal RT have a very high risk of relapse when compared to VWI, CSI, and WBR; relapse risk seems to vary inversely with the volume irradiated.^19^, ^24^ Thus, there seems to be a minimum below which relapse becomes likely. From these data, it is likely WVR using IMRT with a total dose of about 40 Gy is the lowest dose and smallest field that is advisable for an effective treatment that makes relapse highly unlikely. Therefore, WVR with a tumor bed boost is currently the standard for treatment for nonmetastatic GN.^1^ Established radiation dose ranges were followed, with 21–24 Gy for the entire ventricle and a boost directed at the tumor bed for a total dose of 40–45 Gy.^1^, ^37^

With the development of platinum‐based chemotherapy agents, researchers explored combining RT and NC to reduce radiation dose and field. NC can reduce the size of the primary tumor and control microscopic disease^29^ prior to RT. A meta‐analysis comparing RT‐only with NC + RT resulted in a more favorable rate of general survival at 3 years than RT alone, but at 5 years this advantage is eliminated or reverses. The authors of this study recommended favoring the NC + RT regime for patients with pure intracranial GN and severe disease progression.^21^ However, as seen in this case, the patient suffered from acute toxicity from chemotherapy and the dose had to be reduced. Despite this reduced dose, the patient had excellent long‐term local control. This raises the important question of whether lower‐dose NC with good response followed by reduced dose and field RT would have similar treatment outcomes with fewer undesirable side‐effects.

Because of the good prognosis of treatment, and multiple treatment protocols available, it is therefore no surprise that there are several different recommended treatment protocols (Table 3), with some studies supporting a wide‐field NC approach,^19^, ^20^, ^22^, ^23^, ^28^, ^30^, ^31^, ^32^ with others support the use of NC + RT.^1^, ^24^, ^26^, ^29^ However, narrow‐field RT or NC alone have high relapse risks and are not recommended to cure GN.^20^

Therefore, treatment should balance curing the disease and preventing relapse with undesirable sequelae. It is currently unclear whether proposed NC protocols can be reduced in dose and/or number of sessions, while maintaining acceptable long‐term control of GN. For radiation therapy, IMRT WVR with an approximate 45 Gy dose seems to strike this balance, especially if the GN it is staged at M0. It may also be helpful to tune the RT dose depending on the response to NC, with complete response requiring a lower RT dose. This treatment protocol is like those developed for children diagnosed with GN, suggesting that adult GN may be treated using protocols developed for children with little modification. Additional case studies and trials will be necessary to further untangle the effects of different GN treatment protocols.

## CONFLICT OF INTEREST

The authors have stated explicitly that there are no conflicts of interest in connection with this article.

## AUTHOR CONTRIBUTIONS

All authors had full access to the data in the study and take responsibility for the integrity of the data and the accuracy of the data analysis. *Conceptualization*, L.J.F.R., X.M.P.; *investigation*, L.J.F.R., X.M.P.; *data curation*, L.J.F.R., X.M.P.; *writing of this article*, L.J.F.R., X.M.P.

## ETHICAL APPROVAL

The study was approved by the relevant institutional ethics committee. Informed consent for publication was obtained from the patient.

## Data Availability

The data that support the findings of this study are available on request from the corresponding author. The data are not publicly available due to privacy or ethical restrictions.
